# The Effects of Yoga on Pain, Mobility, and Quality of Life in Patients with Knee Osteoarthritis: A Systematic Review

**DOI:** 10.1155/2016/6016532

**Published:** 2016-09-29

**Authors:** Laidi Kan, Jiaqi Zhang, Yonghong Yang, Pu Wang

**Affiliations:** ^1^Rehabilitation Medicine Center, West China Hospital of Sichuan University, Chengdu, China; ^2^West China School of Medicine, Sichuan University, Chengdu, China; ^3^Department of Surgery, Faculty of Medicine, The Chinese University of Hong Kong, Shatin, Hong Kong; ^4^Key Laboratory of Rehabilitation Medicine in Sichuan, Chengdu, China

## Abstract

*Objective.* To systematically assess the effects of yoga on pain, mobility, and quality of life in patients with knee osteoarthritis.* Methods.* Pubmed, Medline, EMBASE, the Cochrane Central Register of Controlled Trials, Physiotherapy Evidence Database (PEDro), and other sources were searched systematically in this study. Two reviewers identified eligible studies and extracted data independently. Downs and Black's Quality Index were used to evaluate the methodological quality of the included studies.* Results.* A total of 9 articles (6 studies) involving 372 patients with knee osteoarthritis met the inclusion criteria. The most common yoga protocol is 40~90 minutes/session, lasting for at least 8 weeks. The effect of yoga on pain relief and function improvement could be seen after two-week intervention.* Conclusion.* This systematic review showed that yoga might have positive effects in relieving pain and mobility on patients with KOA, but the effects on quality of life (QOL) are unclear. Besides, more outcome measure related to mental health of yoga effects on people with KOA should be conducted.

## 1. Introduction

Knee osteoarthritis (KOA) is a degenerative knee disease associated with pain, swelling, stiffness, limited ambulation, and declined balance function [1]. It has been believed that chondrocytes undergo premature aging, which called “stress-induced senescent state” that is the reason for cartilage degeneration [2]. The inflammatory processes, the reduction of lubricin levels, and also the impairments of the synovial fluid lubricating ability, which are closely related to the development of osteoarthritis have also been believed [3]. It is a wear-and-tear arthritis result from the repetitive stress injuries of the joint and sometimes physical damage can make things worse [1, 4]; however, according to the Osteoarthritis Research International (OARSI) guidelines for the nonsurgical management of KOA, exercise was recommended to improve the function and activities participation of people with KOA [5]. The mechanism behind this phenomena may be that physical activity can limit the ameliorating cartilage degeneration by contributing more lubricin expression and decrease the deleterious effects of chondrocyte senescence [6]. In this condition, choosing an appropriate exercise modality is one of the biggest challenges on the field of rehabilitation.

The primary component of exercise training focuses on improving muscles strength; however the balance deficits and stress management are often overlooked [7], which are also other two important factors that affect the mobility of KOA patients. Yoga, as an interesting exercise modality, not only has been proved to have positive effects on physical building [8] but also could give the practitioner a union over their mind, body, and spirit [9, 10], which means yoga may have effects on mental health. The benefits of yoga have been explored in different population [11], including stroke, chronic obstructive pulmonary disease (COPD), and heart disease [8, 12, 13], which prove that yoga may have effect on mood, balance, exercise capacity, and lung function. Besides, yoga has also been used to relieve pain in those with rheumatoid arthritis and chronic low back pain [14, 15]. Some studies have explored the effects of yoga in people with KOA, but no systematic review has stressed that. We conducted this systematic review intentioned to summarize the available evidences on the effect of yoga on people with KOA.

## 2. Methods

### 2.1. Database Sources and Search Strategy

Relevant articles were identified using the following databases: Medline (1966 to Jul 2015; via Ovid), EMBASE (1980 to Jul 2015; via Ovid), the Cochrane Central Register of Controlled Trials (CENTRAL) (The Cochrane Library, Issue 7 of 12 Jul 2015), Pubmed (1966 to Jul 2015), and Physiotherapy Evidence Database (PEDro) (1929 to Jul 2015; via website). Key words included osteoarthritis, knee, yoga, randomized controlled trial, trial, pain, mobility, balance, symptoms, and quality of life. The last search was conducted on December 6, 2015.

### 2.2. Selection Criteria

Articles were considered included when they met the following criteria: (1) studies were published in English; (2) the patients had clear diagnostic criteria of KOA; (3) the intervention type of experimental group is yoga. Articles were excluded if they were (1) patients diagnosed with secondary KOA; (2) animal studies; (3) published as conference processing.

### 2.3. Data Extraction and Quality Assessment

The following pieces of information were extracted from each article: the demographic characteristic of patients, type of study, description of both experimental and control interventions, duration of trial period, and outcome measure. Two authors independently extracted the date and disagreement was resolved by discussion with the third author.

The methodological quality was assessed using the Downs and Black's Quality Index which has well-established validity for both randomized and nonrandomized studies [25]. The Downs and Black's Quality Index has five subscales: (1) clear description of some characteristic; (2) external validity; (3) internal validity; (4) selection bias; and (5) power, which consists of 27 items. The item in power scored 0 to 5 and one item in description scored 0 to 2, and the other items scored 0 or 1; the total score was 32 points. A score of 23 or higher indicates good-quality article with low risk of bias, a score between 22 and 13 indicates medium-quality article with moderate risk of bias, and a score of 12 or lower represents a poor-quality article with high risk of bias. Two reviewers independently assess the quality of article and the disagreements were resolved by the discussion.

## 3. Results

### 3.1. Study Selection

**Figure 1:**
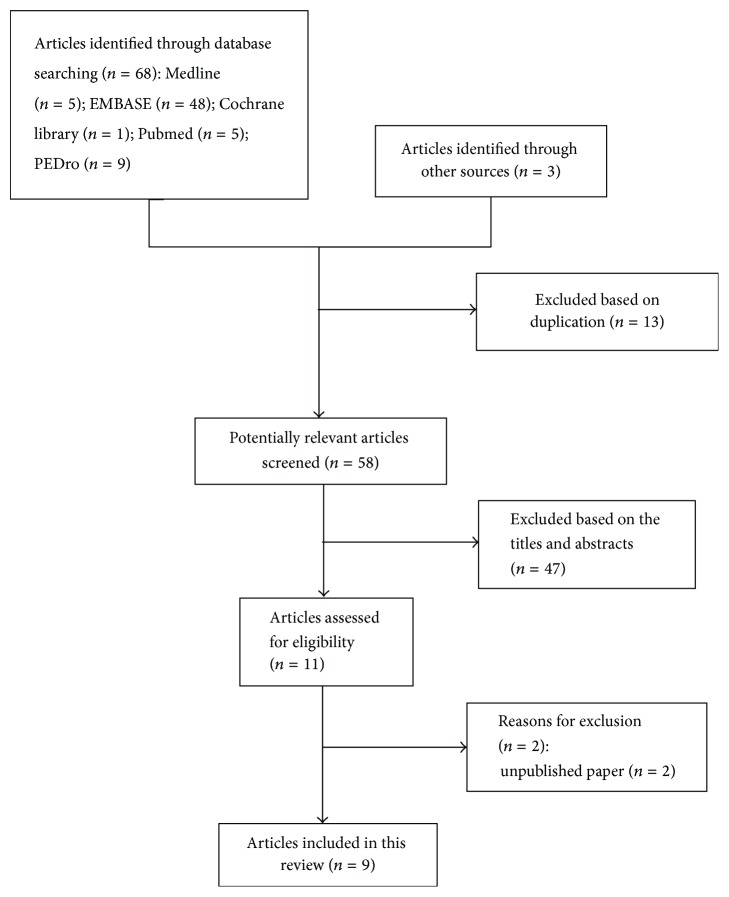
Search strategy and flow chart for this review.

We obtained 71 articles initially, of which 13 articles were excluded for duplication and 47 records were excluded after reading the title and abstracts. After in-depth screening of the remaining 11 articles, two studies were eliminated owing to unpublished conference reports [26, 27]. Finally, 9 articles (6 studies) were selected in this review (Figure 1). Six articles (three RCTs) [16–19, 22, 23], one quasi-RCT [24], and two single group pre-post studies [20, 21] were included.

### 3.2. Participants

The characteristics of 9 (6 studies) articles are given in Table 1. In all studies, 372 subjects were involved; the number of participants in intervention and control group ranged from 11 to 125. The duration of KOA ware required at least 6 months in three studies [19, 23, 24]; only one study had clear description about the duration of KOA [16]. Although the KOA duration of subjects in other two studies has no clear description, they showed consequence in certain symptoms, for example, pain [21, 22]. The mean age of subjects varied from 51 to 71 years. The subjects gender of four studies was all females [19–21, 24]; in one study, the number of males was about half of the females [16], and in other studies the number of males was almost equal to that of females [23].

### 3.3. Quality Assessment

The consequence of quality assessment about the 9 articles (6 studies) is present in Table 2. In view of their score, one of them is considered as good-quality trials (Downs and Black's Quality Index of 23 or higher) [19], seven of them are medium-quality articles (Downs and Black's Quality Index between 22 and 13) [16–18, 20–22, 24], and one trial was poor-quality article (Downs and Black's Quality Index of 12 or lower) [23].

### 3.4. Intervention Characteristics

Among included studies, three of them had control group which does conventional exercise during the experimental time [16, 19, 24], but the control group does yoga exercise similar to yoga group after 8 weeks in one study [19]. In another study, both groups were treated with EMG biofeedback, knee muscle strengthening exercises, and Transcutaneous Electrical Nerve Stimulation (TENS), and the yoga group received additionally Iyengar yoga [23]. Two other studies did not have control group [20, 21].

The yoga group received yoga exercise for 8 weeks in three studies [20, 23, 24]. The experiment time is 20 weeks in one study, but the comparison between two groups just took 8 weeks [19]. 12-week yoga exercise was applied in other two studies [16, 21]. Almost every study had 3-4 sessions per week with each session varying from 60 to 90 minutes. The type of yoga practice in three studies all consisted of* asana* (movement),* pranayama *(breathing), and* meditation* (relaxation) [16, 19, 24], and the type of yoga practiced in other two studies just had* asana* (movement) [20, 23]; the last study did not mention the yoga type but described the subjects posture when doing yoga exercise [21].

## 4. Outcomes

### 4.1. Pain

Two outcome measurements were used to test the pain change in five studies [19–21, 23, 24].

#### 4.1.1. Western Ontario and McMaster Universities OA Index Scale (LK Scale 3.1) (WOMAC)

Two studies used WOMAC as an outcome measure to assess the effects of yoga exercise on pain relief for people with KOA [19, 20]. In Kolasinski et al., the pre- and postintervention scores had significantly improved in pain after 8 weeks of yoga exercise. In Cheung et al. [19], the between-group differences at 8 weeks were significant for pain. There was significant difference in pain in both 4 to 8 weeks' yoga group and 4 to 20 weeks' yoga group [19].

#### 4.1.2. Visual Analog Scale (VAS)

Four studies used VAS assessed pain in people with KOA [16, 21, 23, 24]. In Ebnezar et al. [16], there was a significant difference in pain both within (*p* < 0.001) and between groups (*p* < 0.001) after the 3-month yoga intervention combined with physiotherapy with higher effect size in the yoga group than in the control group (therapeutic exercise with physiotherapy). In Nambi and Shah, yoga group showed a more reduced VAS (56.83%) than control group (38.15%) after 8 weeks of intervention. And the pre- and postintervention ratings of VAS score showed a statistically significant reduction of pain intensity in yoga group compared with control group (*p* < 0.05) [23]. In Brenneman et al. [21], the pre- and postintervention scores had a significant improvement in pain after 12 weeks of yoga-based exercise, and in Ghasemi et al. [24] no significant differences were detected in pain between the 8-week yoga group and the control group (home-based activities); however, the pre-post scores showed a significant difference in the yoga group but not in the control group.

### 4.2. Mobility

Three studies assessed mobility in many ways [16, 20]. In Ebnezar et al. [16], there was a significant difference in walking time within (*p* < 0.001) and between the groups (*p* < 0.001) after 12 weeks of intervention with higher effect size in the yoga than in the control group. But in Kolasinski et al. [20], the 50-foot walk time was unchanged after 8 weeks of yoga exercise. In Brenneman et al. [21], a Six-Minute Walk Test (6MWT), a 30-second chair stand test (30 s CST), and a stair-climbing protocol were used to assesse mobility; the pre- and postintervention scores had a significant improvement when measured with 6MWT (*p* < 0.001) and 30 s CST (*p* = 0.006) after 12 weeks of yoga intervention, but no significant change could be detected in stair-climbing protocol.

### 4.3. Quality of Life

Four studies assessed the quality of life (QOL) as an outcome [17, 19, 21, 24]. Two of them applied Knee Injury and Osteoarthritis Outcome Scale (KOOS) [21, 24]; in Brenneman et al. [21], the pre- and postintervention scores had a significant improvement in QOL after 12 weeks of yoga exercise (*p* < 0.001), and in Ghasemi et al. [24] the pre- and postintervention scores had a significant difference in QOL in the yoga group (*p* < 0.05), but there was no significant difference between the control group and yoga group. In Ebnezar et al., Short Form 36 (SF-36) was used to assess QOL [17]; between and within group differences were highly significant on all domains of the SF-36 (*p* < 0.001) with better improvement in the yoga group than the control group on 15th day and 90th day. And in Cheung et al. they used Short Form Health Survey (SF-12) and Cantril Self-Anchoring Ladder to assess QOL; the Cantril Self-Anchoring Ladder could assess QOL both in “current” times and “in 5 years” times [19]; there was no significant difference between yoga and control group in QOL after 8 weeks of intervention in view of this two assessment scales. During intervention in yoga group, the Cantril Self-Anchoring Ladder “QOL current” scores significantly improved between 4 and 8 weeks (*p* = 0.045), but for “QOL in 5 years” the changes were not significant. No significant improvement was noted in SF-12 over time.

## 5. Discussion 

The purpose of this review is to evaluate the effect of yoga on pain, mobility, and QOL in people with KOA. Although majority of these studies seem to exhibit a favorable effect after yoga intervention, there are still some inconsistent findings in this review.

### 5.1. Pain

Pain is a major symptom for osteoarthritis [1]. The cushioning between joints-cartilage wears away and muscle weakness is considered the major cause of pain and disability. Yoga have been proved to be a positive effect in pain relief in all included studies, which provide some evidence to support the application of yoga as an alternative therapeutic modality in pain management of patients with KOA. Some studies demonstrated that people will achieve better muscle strength and stamina as well as steadiness and flexibility after yoga exercise [28, 29], and, in our included studies, some benefits related to physical functions, like range of motion, and arthritis symptom can be found after yoga intervention, which partly explain the effect on pain relief. The experience of pain is also a psychological phenomenon which has several additional central processes including affective, behavioral, and cognitive factors [30]. However, outcome related to psychological issue is hard to discuss in our included articles. Yoga is considered to be an aerobic exercise combined with breathing training and relaxation therapy and it may have positive effects on pain relief in a comprehensive way, not just in physical but also psychological aspects, so a broaden assessment system for yoga is needed to be established in future studies.

### 5.2. Mobility

The symptoms of KOA, like pain and stiffness, can cause a series of consequences, such as limited ambulation, and worsen quality of life [1]. Yoga exercise had been proved to relieve pain and strengthen muscle strength where both support the fact that yoga may have positive effect on mobility.

Our review show that yoga has positive effect on mobility in two studies [16, 21], but not in Kolasinski et al. [20]. One possible explanation is that the duration of yoga intervention in Kolasinski et al. [20] is not long enough (8 weeks). Also a possible situation is that Kolasinski et al. relatively have larger bias for small sample size (only seven analyzed case). From available evidence [16, 21], 12 weeks of yoga in combination with physical therapy may help improve the short-distance mobility.

Subjects in Kolasinski et al. reported that they had heightened awareness of how they were positioning their bodies in space after yoga intervention [20]. And it seems to improve balance ability to some degree, similar to previous article which reported that yoga had positive effect on balance in people with stroke [8]. But only one study in this review had assessed the balance [19], in which balance ability did not have significant improvement after yoga intervention, but it had a positive effect on repeated chair stands, which means yoga may have positive effects on balance in people with KOA; the most common yoga protocol is 40–90 minutes/session, lasting for at least 8 weeks. But more studies are needed to prove that.

### 5.3. Quality of Life

In addition to a lot of disturbing symptoms that KOA have, the most important thing is that it greatly affects the quality of life of patients with KOA [1]. Quality of life (QOL) is getting more attention to social life [31] and yoga has been proved to have positive effect on Health Related Quality of Life (HRQOL) [7].

The present systematic review showed that yoga intervention has positive effect on QOL based on three studies [17, 21, 24], but Cheung et al. [19] reported an inconsistent result in QOL. However, we think the outcome measure about QOL may have more accurate results if it narrows to HRQOL, which has more reliability in ending change about patients. It seems that yoga have short-term effect on QOL of KOA patients, but more and high quality studies are needed in terms of long-term effects.

In addition to the physical health we discussed above, we believe that the mental health also have a great impact on QOL. Previous studies showed that yoga has positive effect on depression, anxiety, and stress reducing [32, 33]. In our included articles, only Kolasinski and colleagues had description on mental health. It had been assessed using Arthritis Impact Measurement Scale 2 (AIMS 2). Only the AIMS2 Affect Component showed a statistically significant improvement which means yoga may have positive effects on mental health in people with KOA, but more studies and concern are needed for the outcomes of yoga for KOA in mental health.

Yoga may be a safe and tolerable exercise for patients with KOA since no studies reported adverse event both during and after yoga intervention.

## 6. Limitations 

Three limitations could be found in this systematic review. First, we would not conduct quantitative research by performing a meta-analysis because of the heterogeneity of the studies and missing data of some important outcomes. Second, excluded non-English language studies and unpublished articles and conference processing may result in bias. Thirdly, just small amount of RCTs were focused on this area and were included in this review; the lower quality of the studies will limit the power of drawing any conclusion.

## 7. Conclusion 

This systematic review showed that yoga has positive effect on pain relief on people with KOA with good evidence. A relative long period (12 weeks) of yoga intervention may help to improve the short-distance mobility in patients with KOA. More RCTs with high quality and larger sample size are needed. Further work will be needed to address the mechanisms of yoga effect on KOA people and more specific outcomes are needed to concern psychological issues.

## Figures and Tables

**(a):** 

Study	Study design	Number of Participants	Analyzed number of participants	Duration of KOA (yrs)	Age of the participants (mean ± SD)	Gender (F/M)
Ebnezar et al., 2012 [16]	RCT	Y/C = 125/125	Y/C = 118/117	<1 yr/1-2 yrs/>2 yrs = 121/79/50	Y/C = 59.56 ± 9.54/59.42 ± 10.66	174/76

Ebnezar et al., 2012 [17]	RCT	Y/C = 125/125	Y/C = 118/117	<1 yr/1-2 yrs/>2 yrs = 121/79/50	Y/C = 59.56 ± 8.18/59.42 ± 10.66	174/76

Ebnezar and Yogitha, 2012 [18]	RCT	Y/C = 125/125	Y/C = 118/117	Unclear	Y/C = 59.6 ± 8.2/59.4 ± 10.7	Unclear

Cheung et al., 2014 [19]	RCT	Y/C = 18/18	Y/C = 18/18	At least 6 months	Y/C = 71.9 ± 2.7/71.9 ± 3.1	All females

Kolasinski et al., 2005 [20]	Single group pre-post study	Y = 11	Y = 7	At least 6 months	Y = 58.6 ± 8.6	All females

Brenneman et al., 2015 [21]	Single group pre-post study	Y = 45	Y = 39	Unclear	Y = 60.3 ± 6.5	All females

Ebnezar et al., 2012 [22]	RCT	Y/C = 125/125	Y/C = 118/117	<1 yr/1-2 yrs/>2 yrs = 121/79/50	Y/C = 59.6 ± 8.2/59.4 ± 10.7	174/76

Nambi and Shah, 2013 [23]	RCT	Y/C = 15/15	Y/C = 15/15	At least 6 months	Y/C = 52 ± 5/54 ± 4	13/17

Ghasemi et al., 2013 [24]	Quasi-RCT	Y/C = 15/15	Y/C = 11/14	Unclear	Y/C = 51 ± 8.9/53.11 ± 10.9	All females

**(b):** 

Study	Comparison Intervention	Intervention of control group	Intervention of yoga group	Yoga therapy practice	Main outcomes	Time point
Ebnezar et al., 2012 [16]	Yoga + PT versus PT	PT (20 minutes/day/2 weeks) Practices (40 minutes/day) Home practice (12 weeks) Compliance (once/3 days) Weekly review (once/week/12 weeks)	PT (20 minutes/day/2 weeks) Integrated yoga therapy (40 minutes/day/2 weeks) Integrated yoga therapy (40 minutes/day/10 weeks)	Yogic sukshma vyayamas Relaxation techniques Asanas (physical postures) Pranayama Meditation Lectures and counseling	Walking pain Walking time (50 m) WOMAC Sign: active range of movements Sign: tenderness Sign: swelling Sign: crepitus	14 weeks

Ebnezar et al., 2012 [17]	Yoga + PT versus PT	PT (20 minutes/day/2 weeks) Practices (40 minutes/day; 6 days/week) Home practice (12 weeks) Compliance (once/3 days) Weekly review (once/week/12 weeks)	PT (20 minutes/day/2 weeks) Integrated yoga therapy (40 minutes/day/2 weeks) Integrated yoga therapy (40 minutes/day/10 weeks)	Yogic sukshma vyayamas Relaxation techniques Asanas (physical postures) Pranayama Meditation Lectures and counseling	QOL (SF-36)	14 weeks

Ebnezar and Yogitha, 2012 [18]	Yoga + PT versus PT	PT (20 minutes/day/2 weeks) Practices (40 minutes/day) Home practice (12 weeks) Compliance (once/3 days) Weekly review (once/week/12 weeks)	PT (20 minutes/day/2 weeks) Integrated yoga therapy (40 minutes/day/2 weeks) Integrated yoga therapy (40 minutes/day/10 weeks)	Yogic sukshma vyayamas Relaxation techniques Asanas (physical postures) Pranayama Meditation Lectures and counseling	Walking pain WOMAC Sign: tenderness Sign: early morning stiffness	14 weeks

Cheung et al., 2014 [19]	Yoga versus Usual care (8 weeks) Preyoga intervention versus postyoga intervention (8 weeks–20 weeks)	Another program (8 weeks) Hatha yoga intervention (8–20 weeks)	Hatha yoga (60 minutes/week/8 weeks) Home practice yoga (30 minutes/time; 4 times/week)	Asanas Pranas Meditation	WOMAC SPPB PSQI QOL (SF-12 & Cantril Self-Anchoring Ladder)	20 weeks

Kolasinski et al., 2005 [20]	Yoga versus control (no specific exercise)		Modified Iyengar yoga (90-minute classes/week/8 weeks)	Asanas	WOMAC AIMS2 GA Psychological subsets Physician Global Assessment 50-foot walk time	8 weeks

Brenneman et al., 2015 [21]	Yoga versus control (no specific exercise)		Yoga (60 minutes/sessions/3 sessions/week/12 weeks)	Unclear	VAS KOOS Fitness and peak KAM Strength Mobility performance	12 weeks

Ebnezar et al., 2012 [22]	Yoga + PT versus PT	PT (20 minutes/day/2 weeks) Practices (40 minutes/day) Home practice (12 weeks) Compliance (once/3 days) Weekly review (once/week/12 weeks)	PT (20 minutes/day/2 weeks) Integrated yoga therapy (40 minutes/day/2 weeks) Integrated yoga therapy (40 minutes/day/10 weeks)	Yogic sukshma vyayamas Relaxation techniques Asanas (physical postures) Pranayama Meditation Lectures and Counseling	Anxiety scores Resting pain Sign: early morning stiffness	14 weeks

Nambi and Shah, 2013 [23]	Yoga + EMG biofeedback + Knee strengthening exercise + TENS versus EMG biofeedback + Knee strengthening exercise + TENS	EMG biofeedback (3 times/week/8 weeks) Knee strengthening exercise (3 times/week/8 weeks) TENS (20 minutes/time/3 times/week)	Iyengar yoga (90 minutes/session, 3 times/week/8 weeks) EMG biofeedback (3 times/week/8 weeks) Knee strengthening exercise (3 times/week/8 weeks) TENS (20 minutes/time/3 times/week)	Asanas	VAS WOMAC	8 weeks

Ghasemi et al., 2013 [24]	Yoga versus ordinary daily activities	Ordinary daily activities	Hatha yoga (60 minutes/session, 3 times/week/8 weeks)	*Asana* (movement) *Pranayama* (breathing) Meditation (relaxation)	VAS KOOS	8 weeks

WOMAC: Western Ontario and McMaster Universities Osteoarthritis Index (lower scores = better state); SPPB: Short Physical Performance Battery (higher scores = better state); QOS: quality of sleep; QOL: quality of life; PSQI: Pittsburgh Sleep Quality Index (lower scores = better state); SF-12: Health Related Short Form 12 (higher scores = better state); PCS: physical component summary; MCS: mental component summary; Cantril current and 5 years (higher scores = better state); AIMS2: Arthritis Impact Measurement Scale 2; GA: Global Assessment; ADL: Activities of Daily Life; VAS: Visual Analog Scale; KOOS: Knee Injury and Osteoarthritis Outcome Scale; KAM: knee adduction moment; PT: physiotherapy; EMG: electromyography.

**Table 2:** Quality of article included in this review using Downs and Black scale.

Measures	Ebnezar et al., 2012 [16]	Ebnezar et al., 2012 [17]	Ebnezar and Yogitha, 2012 [18]	Cheung et al., 2014 [19]	Kolasinski et al., 2005 [20]	Brenneman et al., 2015 [21]	Ebnezar et al., 2012 [22]	Nambi and Shah, 2013 [23]	Ghasemi et al., 2013 [24]
*Clear description of the following?*									
(1) Hypothesis/aim/objective	1	1	1	1	1	1	1	1	1
(2) Main outcome	1	1	1	1	1	1	1	1	1
(3) Characteristics of participants	1	1	1	1	1	1	1	1	1
(4) Intervention of interest	1	1	1	1	1	1	1	1	1
(5) Distribution of principal confounders in each group	0	0	0	0	0	0	0	0	2
(6) Main findings	1	1	1	1	1	1	1	1	1
(7) Estimates of random variability for main outcomes	1	1	1	1	1	1	1	1	1
(8) All important adverse events that may be a consequence of intervention	0	0	0	1	1	0	0	1	0
(9) Characteristics of patients lost to follow-up	1	1	1	1	1	1	1	0	0
(10) Actual probability values for main outcomes	1	1	1	1	1	1	1	1	1
*External validity*									
(11) Were invitees representative of the population from which they were recruited?	0	0	0	1	0	0	0	0	0
(12) Were subjects who were prepared to participate representative of the population from which recruited?	0	0	0	1	0	1	0	0	0
(13) Were the staff, places, and facilities representative of the treatment that the majority of subjects received?	0	0	0	1	1	1	0	0	0
*Internal validity*									
(14) Was an attempt made to blind subjects to the intervention they received?	0	0	0	0	0	0	0	0	0
(15) Was an attempt made to blind those measuring main outcomes of the intervention?	0	0	0	1	0	0	0	0	0
(16) If any results were based on “data dredging,” was this made clear?	0	0	0	1	1	1	0	1	1
(17) In trials and cohort studies, did analyses adjust for length of follow-up? Or, in case-control studies, was the period between intervention and outcome the same for cases and controls?	1	1	1	1	1	1	1	1	0
(18) Were appropriate statistical tests used to assess the main outcomes?	1	1	1	1	1	1	1	0	1
(19) Was compliance with the intervention reliable?	1	1	1	1	1	1	1	1	1
(20) Were main outcome measures reliable and valid?	1	1	1	1	1	1	1	0	1
*Internal validity-confounding (selection bias)*									
(21) For trials and cohort studies, were patients in different intervention groups? For case-control studies, were cases and controls recruited from the same population?	1	1	1	1	1	1	1	0	0
(22) For trials and cohort studies, were subjects in different intervention groups? For case-control studies, were cases and controls recruited over same period of time?	1	1	1	1	1	0	1	0	0
(23) Were subjects randomized to intervention groups?	1	1	1	1	0	0	1	0	0
(24) Was the randomized intervention assignment concealed from both patients and staff until recruitment was complete? Was it irrevocable?	1	1	1	1	0	0	1	0	0
(25) Was there adequate adjustment for confounding in analyses from which main findings were drawn?	0	0	0	0	0	0	0	0	0
(26) Were losses of subjects to follow-up taken into account?	1	1	1	1	1	0	1	0	0
*Power *									
(27) Was there sufficient power to detect a clinically important effect when *p* < 0.05?	0	0	0	0	0	0	0	0	0
Total score (maximum 32)	17	17	17	23	18	16	17	11	13

A score of 23 or higher indicates good-quality article with low risk of bias.

A score between 22 and 13 indicates medium-quality article with moderate risk of bias.

A score of 12 or lower represents a poor-quality article with high risk of bias [25].

## References

[1] Zelman D. Osteoarthritis of the knee (Degenerative arthritis of the knee). http://www.webmd.com/osteoarthritis/guide/ostearthritis-of-the-knee-degenerative-arthritis-of-the-knee?page=2.

[2] Musumeci G., Mobasheri A., Szychlinska M. A. (2015). Age-related degeneration of articular cartilage in the pathogenesis of osteoarthritis: molecular markers of senescent chondrocytes. *Histology and Histopathology*.

[3] Szychlinska M., Leonardi R., Al-Qahtani M., Mobasheri A., Musumeci G. (2016). Altered joint tribology in osteoarthritis: reduced lubricin synthesis due to the inflammatory process. New horizons for therapeutic approaches. *Annals of Physical and Rehabilitation Medicine*.

[4] Fransen M., McConnell S., Harmer A. R., Van der Esch M., Simic M., Bennell K. L. (2015). Exercise for osteoarthritis of the knee. *Cochrane Database of Systematic Reviews*.

[5] McAlindon T. E., Bannuru R. R., Sullivan M. C. (2014). OARSI guidelines for the non-surgical management of knee osteoarthritis. *Osteoarthritis and Cartilage*.

[6] Musumeci G., Castrogiovanni P., Trovato F. M. (2015). Physical activity ameliorates cartilage degeneration in a rat model of aging: a study on lubricin expression. *Scandinavian Journal of Medicine and Science in Sports*.

[7] Desveaux L., Lee A., Goldstein R., Brooks D. (2015). Yoga in the management of chronic disease. *Medical Care*.

[8] Lazaridou A., Philbrook P., Tzika A. A. (2013). Yoga and mindfulness as therapeutic interventions for stroke rehabilitation: a systematic review. *Evidence-Based Complementary and Alternative Medicine*.

[9] Ernst E., Pittler M. H., Wider B. (2008). *Oxford Handbook of Complementary Medicine*.

[10] Kappmeier K. L., Ambrosini D. M. (2006). *Instructing Hatha Yoga*.

[11] Saper R. B., Eisenberg D. M., Davis R. B., Culpepper L., Phillips R. S. (2004). Prevalence and patterns of adult yoga use in the United States: results of a national survey. *Alternative Therapies in Health and Medicine*.

[12] Liu X.-C., Pan L., Hu Q., Dong W.-P., Yan J.-H., Dong L. (2014). Effects of yoga training in patients with chronic obstructive pulmonary disease: a systematic review and meta-analysis. *Journal of Thoracic Disease*.

[13] Cramer H., Lauche R., Haller H., Dobos G., Michalsen A. (2015). A systematic review of yoga for heart disease. *European Journal of Preventive Cardiology*.

[14] Bosch P. R., Traustadóttir T., Howard P., Matt K. S. (2009). Functional and physiological effects of yoga in women with rheumatoid arthritis: a pilot study. *Alternative Therapies in Health and Medicine*.

[15] Groessl E. J., Weingart K. R., Aschbacher K., Pada L., Baxi S. (2008). Yoga for veterans with chronic low-back pain. *Journal of Alternative and Complementary Medicine*.

[16] Ebnezar J., Nagarathna R., Yogitha B., Nagendra H. R. (2012). Effects of an integrated approach of hatha yoga therapy on functional disability, pain, and flexibility in osteoarthritis of the knee joint: a randomized controlled study. *Journal of Alternative and Complementary Medicine*.

[17] Ebnezar J., Nagarathna R., Yogitha B. B. (2012). Effects of an integrated approach of hatha yoga therapy on quality of life in osteoarthritis of the knee joint: a randomized control study. *International Journal of Yoga*.

[18] Ebnezar J., Yogitha B. (2012). Effectiveness of yoga therapy with the therapeutic exercises on walking pain, tenderness, early morning stiffness and disability in osteoarthritis of the knee joint—a comparative study. *Journal of Yoga & Physical Therapy*.

[19] Cheung C., Wyman J. F., Resnick B., Savik K. (2014). Yoga for managing knee osteoarthritis in older women: a pilot randomized controlled trial. *BMC Complementary and Alternative Medicine*.

[20] Kolasinski S. L., Garfinkel M., Tsai A. G., Matz W., Van Dyke A., Schumacher H. R. (2005). Iyengar yoga for treating symptoms of osteoarthritis of the knees: A Pilot Study. *Journal of Alternative and Complementary Medicine*.

[21] Brenneman E. C., Kuntz A. B., Wiebenga E. G., Maly M. R. (2015). A yoga strengthening program designed to minimize the knee adduction moment for women with knee osteoarthritis: a proof-of-principle cohort study. *PLoS ONE*.

[22] Ebnezar J., Nagarathna R., Yogitha B., Nagendra H. R. (2012). Effect of integrated yoga therapy on pain, morning stiffness and anxiety in osteoarthritis of the knee joint: a randomized control study. *International Journal of Yoga*.

[23] Nambi G. S., Shah A. K. (2013). Additional effect of iyengar yoga and EMG biofeedback on pain and functional disability in chronic unilateral knee osteoarthritis. *International Journal of Yoga*.

[24] Ghasemi G. A., Golkar A., Marandi S. M. (2013). Effects of *Hata* yoga on knee osteoarthritis. *International Journal of Preventive Medicine*.

[25] Downs S. H., Black N. (1998). The feasibility of creating a checklist for the assessment of the methodological quality both of randomised and non-randomised studies of health care interventions. *Journal of Epidemiology and Community Health*.

[26] Srivastava R. N., Avasthi V., Srivastava S. R., Raj S. (2015). Does yoga improve pain, stiffness and physical disability in knee osteoarthritis?—a randomize controlled clinical trial. *Osteoarthritis and Cartilage*.

[27] Cheung C., Wyman J., Resnick B. (2012). Is yoga effective for knee osteoarthritis in older women?. *Osteoarthritis Cartilage*.

[28] Dash M., Telles S. (2001). Improvement in hand grip strength in normal volunteers and rheumatoid arthritis patients following yoga training. *Indian Journal of Physiology and Pharmacology*.

[29] Raghuraj P., Telles S. (1997). Muscle power, dexterity skill and visual perception in community home girls trained in yoga or sports and in regular school girls. *Indian Journal of Physiology and Pharmacology*.

[30] Burton D. G. A., Allen M. C., Bird J. L. E., Faragher R. G. A. (2005). Bridging the gap: ageing, pharmacokinetics and pharmacodynamics. *The Journal of Pharmacy and Pharmacology*.

[31] Nussbaum M., Sen A. (1993). *The Quality of Life*.

[32] Cabral P., Meyer H. B., Ames D. (2011). Effectiveness of yoga therapy as a complementary treatment for major psychiatric disorders: a meta-analysis. *The Primary Care Companion for CNS Disorders*.

[33] Ross A., Thomas S. (2010). The health benefits of yoga and exercise: a review of comparison studies. *Journal of Alternative and Complementary Medicine*.

